# A Importância dos Programas de Exercícios Pós-Infarto

**DOI:** 10.36660/abc.20210109

**Published:** 2021-04-08

**Affiliations:** 1 Universidade Federal Fluminense Medicina Clínica NiteróiRJ Brasil Universidade Federal Fluminense - Medicina Clínica, Niterói, RJ - Brasil.; 2 Fit Labor Centro de Performance Humana NiteróiRJ Brasil Fit Labor Centro de Performance Humana, Niterói, RJ - Brasil.

**Keywords:** Infarto do Miocárdio/mortalidade, Exercício, Reabilitação Cardíaca, Atividade Física, Ratos, Disfunção Ventricular, Ecocardiografia/métodos

A atividade física e os exercícios podem reduzir o risco de mortalidade cardiovascular na população em geral em 30%–50%, e a mortalidade por qualquer causa em 20%–50%.^1^ Em pacientes cardíacos, para cada incremento de 1 Met (3,5 mL O_2_ kg^−1^ min^−1^) na capacidade funcional (CF) obtida em um programa de reabilitação cardíaca (PRC), temos uma redução na mortalidade geral de até 13%.^1^^,^^2^ A atividade física e os exercícios reduzem a hospitalização global em 18% e melhoram a qualidade de vida (QV) nessa população.^2^^,^^3^ Após a angioplastia, o PRC resulta em uma redução de 20% nos eventos cardíacos e no número de hospitalizações em comparação com os indivíduos que permaneceram sedentários.^1^^,^^2^^,^^4^

Nesse contexto, os PCRs têm se consolidado como uma estratégia terapêutica segura, que ameniza os efeitos do descondicionamento físico progressivo decorrente das doenças cardiovasculares (DC). O exercício bem orientado é a pedra angular no manejo da DC e seus principais fatores de risco.^1^^–^^6^ Os PRCs, principalmente na síndrome coronariana pós-aguda (SCA) e em pacientes com disfunção ventricular, trazem importantes benefícios de impacto clínico, com nível de evidência IA nessa população, referenciado por diversos consensos, metanálises e diretrizes.^1^^–^^4^^,^^6^^,^^7^

A importância de se iniciar um PCR, com ênfase no treino aeróbio (TA), logo após a estabilização de um exame SCAg, é revisada em diversos artigos e metanálises.^1^^–^^3^^,^^6^^–^^8^ O TA está associado a menor expressão de receptores beta-adrenérgicos, que predizem o prognóstico em pacientes com maior área de infarto. Melhora diversas variáveis relacionadas ao prognóstico e CF, além dos parâmetros ecocardiográficos (ECO) da remodelação ventricular e biomarcadores.^8^^–^^10^

A literatura menciona inúmeros efeitos benéficos, não apenas sistêmicos, mas principalmente efeitos cardioprotetores^1^^–^^4^^,^^6^^,^^8^^,^^11^ Précoma et al.^3^ e Fletcher et al.^4^ listam esses efeitos, mas aqui iremos destacar um da hemodinâmica: remodelação cardíaca.

O infarto agudo do miocárdio (IAM) pode induzir alterações na geometria ventricular, levando a remodelação ventricular adversa.^8^^–^^10^^,^^12^ Essa alteração na geometria ventricular é o principal fator para o desenvolvimento futuro da disfunção ventricular, apesar dos avanços na revascularização e nas terapias medicamentosas.^9^ A remodelação ventricular esquerda (VE) é um preditor preciso de mortalidade cardíaca após IAM,^8^^–^^10^^,^^12^ mas não está claro como o exercício afeta esse processo. Haykowsky et al.,^9^ em sua metanálise, analisam esse efeito, mostrando resultados diferentes. Eles verificaram que, embora existam efeitos benéficos na remodelação ventricular, eles se baseiam nas características da população, na modalidade e na variação na prescrição de exercícios e intervenções, não sendo possível definir por que essas variações ocorrem.

Compreender essas incongruências e os efeitos do exercício na remodelação VE é importante, pois esse conhecimento pode ser usado para aumentar os benefícios do exercício após o IAM.

Um artigo de Souza et al.^13^ analisa os efeitos tardios do TA no pós-infarto tardio em modelos animais. Isso representa mais uma tentativa de esclarecer essa questão.

Em estudo elegante e controlado, Souza et al.^13^ induziram infarto do miocárdio (IM) ligando a artéria coronária descendente anterior esquerda. Três meses depois, os ratos sobreviventes foram submetidos a ECO transtorácico e teste de esforço, sendo então divididos em três grupos: grupo controle com animais submetidos à cirurgia Sham (Sham n=15); grupo IM sedentários (IM-SED, n=22) e grupo IM exercício aeróbio (IM-EA, n=21) por três meses. Os autores avaliaram a influência do EA na CF, estruturas cardíacas, função VE e expressão do gene da subunidade NADPH (nicotinamida adenina dinucleotídeo fosfato) oxidase em ratos com IM de pequenas dimensões.

Os autores usaram um protocolo de EA de intensidade moderada. Observaram que o exercício era seguro e que o grupo IM-EA alcançou maior tempo de esteira e distância percorrida do que os grupos IM-SED e submetido à cirurgia Sham. Os resultados da cirurgia Sham incluíram redução da CF causada por um estilo de vida sedentário. Apesar de melhorar o desempenho funcional, os efeitos do EA na remodelação cardíaca não foram substanciais em ratos com infarto do miocárdio de pequenas dimensões. Mas o EA foi útil para preservar a geometria do VE, pois a relação entre a espessura da parede posterior diastólica do VE e o diâmetro diastólico do VE mostrou-se reduzida no grupo IM-SED e preservada no grupo IM-EA. A expressão do gene da subunidade NADPH oxidase, importante fonte de geração de espécies reativas de oxigênio, não esteve envolvida na remodelação cardíaca observada em ratos com infarto de pequenas dimensões.

Pela primeira vez, o estudo mostra que o EA tardio, iniciado três meses após o IM, quando a remodelação cardíaca está estável, atenua as alterações da geometria cardíaca em ratos com infarto de pequenas dimensões. Os autores reforçam o conceito do possível benefício da reabilitação cardíaca após SCA, independentemente do grau da lesão cardíaca.
