# Supplementary material for: A Importância dos Programas de Exercícios Pós-Infarto
Source: Arq Bras Cardiol. 2021 Apr 8;116(4):793–4. [Article in Portuguese] doi: 10.36660/abc.20210109 (PMC8121403; doi:10.36660/abc.20210109)
Supplement: Translation

# Translation: The Importance of Post-Infarction Exercise Programs

**Affiliations:** 1 NiteróiRJ Brazil Universidade Federal Fluminense - Medicina Clínica, Niterói, RJ - Brazil.; 2 NiteróiRJ Brazil Fit Labor Centro de Performance Humana, Niterói, RJ - Brazil.

**Keywords:** Myocardial Infarction/mortality, Exercise, Cardiac, Rehabilitation, Physical Activity, Rats, Ventricular Dysfunction, Echocardiography/methods

Physical activity and exercising are said to reduce the risk of cardiovascular mortality in the general population by 30%–50%, and mortality from any cause by 20%–50%.^1^ In cardiac patients, for each 1 Met increment (3.5 mL O_2_ kg^−1^ min^−1^) in functional capacity (FC) obtained in a cardiac rehabilitation program (CRP), we have a reduction in overall mortality of up to 13%.^1^^,^^2^ They reduce global hospitalization by 18%, and improve quality of life (QoL) in this population.^2^^,^^3^ After an angioplasty, CRP results in a 20% reduction in cardiac events and in the number of hospitalizations compared to individuals who remained sedentary.^1^^,^^2^^,^^4^

In this context, CRPs have established themselves as a safe therapeutic strategy, which mitigates the effects of progressive physical deconditioning resulting from cardiovascular diseases (CD). Well-oriented exercise is the cornerstone in handling CD and its main risk factors.^1^^–^^6^ CRPs, especially in post-acute coronary syndrome (ACS) and patients with ventricular dysfunction, bring important benefits of clinical impact, with an evidence level IA in this population, referenced by numerous consensuses, meta analyses and guidelines.^1^^–^^4^^,^^6^^,^^7^

The importance of starting a CRP, with an emphasis on aerobic training (AT), right after the stabilization of an ACS (acute coronary syndrome), is reviewed in different articles and meta analyses.^1^^–^^3^^,^^6^^–^^8^ AT is associated with a lower expression of beta-adrenergic receptors, which predict prognosis in patients with a larger infarcted area. It improves several variables related to prognosis and FC, in addition to echocardiographic parameters (ECHO) of ventricular remodeling, and biomarkers.^8^^,^^9^^,^^10^

The literature mentions numerous beneficial effects, not only systemic, but mainly cardioprotective effects.^1^^–^^4^^,^^6^^,^^8^^,^^11^ Précoma et al.^3^ and Fletcher et al.^4^ list these effects, but here we will highlight one of the hemodynamics: cardiac remodeling.

Acute myocardial infarction (AMI) can induce changes in ventricular geometry, leading to adverse ventricular remodeling.^8^^–^^10^^,^^12^ This change in ventricular geometry is the main contributor to the future development of ventricular dysfunction, despite advances in revascularization and drug therapies.^9^ Left ventricular (LV) remodeling is an accurate predictor of cardiac mortality after AMI,^8^^–^^10^^,^^12^ but it is not clear how exercise affects this process. Haykowsky et al.,^9^ in their meta-analysis, analyze this effect, showing different results. They have found that, although the beneficial effects on ventricular remodeling exist, they are based on population characteristics, modality, and variation in the prescription of exercises and interventions, and it is not possible to define why these variations occur.

Understanding these inconsistencies and the effects of exercise on LV remodeling is important, as this knowledge can be used to increase the benefits of exercise after AMI.

An article by Souza et al.^13^ analyzes the late effects of AT in late post-infarction in animal models. This represents yet another attempt to clarify this issue.

In an elegant, controlled study, Souza et al.^13^ induced myocardial infarction (MI) by ligating the left anterior descending coronary artery. Three months later, surviving rats were subjected to transthoracic ECHO and exercise testing, then were assigned to three groups: sham-operated animals were used as controls (Sham n=15); sedentary MI (MI-SED, n=22) and aerobic exercised MI (MI-AE, n=21) for three months. They evaluated the influence of AE on FC, cardiac structures, LV function, and NADPH (nicotinamide adenine dinucleotide phosphate) oxidase subunit gene expression in rats with small-sized MI.

The authors used a moderate intensity AE protocol. They observed that exercise was safe, and the MI-AE group attained a higher treadmill time and distance run than the MI-SED and Sham-operated groups. The sham surgery results included reduced FC caused by a sedentary lifestyle. Despite improving functional performance, the effects of AE on cardiac remodeling were not substantial in small-sized myocardial infarction rats. But AE was helpful in preserving LV geometry, as the relationship between LV diastolic posterior wall thickness and LV diastolic diameter was reduced in the MI-SED group and preserved in the MI-AE group. The NADPH oxidase subunit gene expression, an important source of reactive oxygen species generation, was not involved in the cardiac remodeling observed in rats with small-sized infarction.

For the first time, the study shows that late AE, initiated three months after MI, when cardiac remodeling is stable, attenuates cardiac geometry changes in rats with small-sized infarction. The authors reinforce the concept of the potential benefit from cardiac rehabilitation after ACS regardless of the degree of cardiac injury.
